# Exploring health researchers’ perceptions of policymaking in Argentina: a qualitative study

**DOI:** 10.1093/heapol/czu071

**Published:** 2014-09-11

**Authors:** Adrijana Corluka, Adnan A Hyder, Peter J Winch, Elsa Segura

**Affiliations:** ^1^Department of International Health, Johns Hopkins Bloomberg School of Public Health, 615 North Wolfe Street, Baltimore, MD 21205, USA and ^2^National Institute of Parasitology, “Dr. M. Fatala Chabén”, Administración Nacional de Laboratorios e Institutos de Salud (ANLIS), Ministerio de Salud, Buenos Aires, Argentina

**Keywords:** Argentina, evidence-based policymaking, health research systems, health researchers, interviews, qualitative, research-to-policy

## Abstract

Much of the published research on evidence-informed health policymaking in low- and middle-income countries has focused on policymakers, overlooking the role of health researchers in the research-to-policy process. Through 20 semi-structured, in-depth qualitative interviews conducted with researchers in Argentina’s rural northwest and the capital of Buenos Aires, we explore the perspectives, experiences and attitudes of Argentine health researchers regarding the use and impact of health research in policymaking in Argentina. We find that the researcher, and the researcher’s function of generating evidence, is nested within a broader complex system that influences the researcher’s interaction with policymaking. This system comprises communities of practice, government departments/civil society organizations, bureaucratic processes and political governance and executive leadership. At the individual level, researcher capacity and determinants of research availability also play a role in contributing to evidence-informed policymaking. In addition, we find a recurrent theme around ‘lack of trust’ and explore the role of trust within a research system, finding that researchers’ distrust towards policymakers and even other researchers are linked inextricably to the sociopolitical history of Argentina, which contributes to shaping researchers’ identities in opposition to policymakers. For policymakers, national research councils and funders of national health research systems, this article provides a deeper understanding of researchers’ perceptions which can help inform and improve programme design when developing interventions to enhance research utilization and develop equitable and rational health policies. For donors and development agencies interested in health research capacity building and achieving development goals, this research demonstrates a need for investment in building research capacity and training health researchers to interact with the public policy ‘world’ and enhancing research communications and transferability to decision makers. It also highlights an opportunity to invest in implementation research platforms, such as health policy research and analysis institutions.

**KEY MESSAGES**

- Barriers and facilitators to evidence-informing policy, as identified by Argentine health researchers, were organized in a nested hierarchical model which includes political governance, bureaucratic processes, institutions, communities of practice and, at the individual level, researcher capacity and research availability.
- Trust, determinants of research availability and the Argentine sociopolitical context are important considerations for evidence-informed policymaking.
- Where applicable, researchers should clearly outline the policy implications of their research so as to facilitate policymakers’ use of the research results.
- For donors and development agencies interested in health research capacity building, this research demonstrates a need for investment in building capacity and training health researchers to interact with the public policy ‘world’ and enhancing research communications and transferability to decision makers. It also highlights an opportunity to invest in implementation research platforms, such as health policy research and analysis institutions.

‘You are in public health and public health is political’. (Informant 18)

‘Argentina is a contradictory country. It is a contradictory country’. (Informant 5)

## Introduction

In the research-to-policy spectrum, much attention has been focused on the policymakers (Innvaer *et al.* 2002; Aaserud *et al.* 2005; Fickel and Thrush 2005; Tomson *et al.* 2005; Dobbins *et al.* 2009). They are considered to play a large role in determining ‘pull factors’ and in creating user demand for research to inform policy (Oxman *et al.* 2009).

Also important is the role of ‘researcher push’ (van Kammen *et al.* 2006). Previous studies have shown that policymakers and health professionals consider ‘home-grown’ research to have greater weight than research results coming from other countries (Hanney *et al.* 2003; Guindon *et al.* 2010). However, the involvement of researchers in the policy process is little studied, particularly in developing countries (Tomson *et al.* 2005). Researchers have only recently begun to study civil society (Cliff *et al.* 2010) and health researchers (Dagenais *et al.* 2009; Cameron *et al.* 2010). For instance, in the first published questionnaire of its kind developed for low- and middle-income countries (LMICs), Cameron *et al.* (2010) tested a questionnaire for researchers in 10 LMICs focused on researchers’ engagement in bridging activities related to high-priority topics (Cameron *et al.* 2010). And in one of the first studies on researchers’ influence on decision makers in Mexico, in 1994, Trostle *et al.* (1999) interviewed both researchers and decision makers about the relationship between health research and policy in the programme areas of AIDS, cholera, family planning and immunization (Trostle *et al.* 1999). Research on national health research systems is gaining greater recognition and importance in the research-to-policy field, with recent posited conceptual models placing researchers as the central stakeholder (Hanney and Gonzalez-Block 2009). There are increasing pressures on countries to base their programming and policy decisions on research/evidence; however, we lack information about what opportunities exist for local researchers to inform policy in Argentina.

The goal of this study was to describe the perspectives of Argentine health researchers regarding evidence-based policymaking writ large, as well as health researchers’ perspectives on the facilitators and barriers to evidence-informed policymaking in Argentina. This research focuses on the supply side of the research-to-policy spectrum in Argentina, explores researchers’ roles in evidence-informed decision making and proposes a new framework for thinking about how researchers interact with (and can influence) their working environment.

## Methods

Semi-structured, in-depth interviews were conducted face-to-face with 20 key informant health research participants in the Federal City of Buenos Aires and the provinces of Salta, Jujuy, Tucuman, Santiago del Estero and Catamarca. Informants were recruited based on contacts provided by Argentine collaborators and by doing both purposive and snowball sampling (Green and Thorogood 2009). All 20 respondents were identified to us as researchers and self-identified as researchers, and came from a wide variety of biological and social science academic backgrounds. Informants were working as researchers in universities, in a combined research and decision-making capacity for provincial Ministries of Health, or within non-governmental organizations (NGOs), such as think tanks. Two individuals interviewed were researchers who were former Ministers of Health at the provincial level.

An interview guide was developed based on a previous complementary study where policymakers were interviewed (Hyder *et al.* 2011), and adapted to reflect the Argentine health research context (Appendix 1). Researchers were asked about their experiences in informing health policies or programmes with their research, and in working with policymakers in the Argentine public health sector, irrespective of content area or local, provincial or national scale. They were also asked what their perceptions were of policymakers and the policymaking process in Argentina, as well as what they perceived to be facilitators of or barriers to research use in policymaking.

All interviews were conducted by one individual (AC), with 18 individuals interviewed one-on-one, and one interview with two individuals. The interviews lasted between 33 min and 1 h 49 min and were completed between May and August 2008. Detailed notes were taken during the interviews and all interviews were recorded on a digital voice recorder. Interviews were transcribed from the original Spanish by a professional transcriber, except for three interviews which were conducted in English and transcribed directly by the interviewer. ATLAS.ti software was used to store and analyse the data. Data were analysed by one individual (AC).

This study takes a constructivist epistemological approach, where knowledge and the human condition are dependent on perception and experience. We drew on aspects of grounded theory (Glaser and Strauss 1967; Charmaz 2001) such as coding, memo writing and reflexivity, whereby the researcher self-scrutinizes decisions, biases and how the researcher has affected the research process and inquiry and ultimately our findings (Moon 2008). Transcripts were analysed by inductive thematic coding and memo-writing. Theoretical saturation was reached prior to all the interviews being completed.

Argentine health researchers were defined as individuals who self-identified as spending more than 50% of their time doing research related to human health and disease. These included, e.g. laboratory researchers, health economists, sociologists and epidemiologists. It was important to recruit health researchers working in fields of research, such as Chagas disease research, which could practically inform policy and legislation.

Health policymakers, as defined in this study, included health policy advisors, bureaucrats and those who prepare briefing notes and policy documents that in turn help inform and shape the final decision-makers’ policy determination/ruling. Researchers understood ‘policymakers’ to be interchangeable with ‘politicians’ (both of which are called ‘politicos’ in Spanish) and were also synonymous with ‘decision makers’ (known as ‘decisores’ or ‘decisores políticos’).

## Results

**Figure 1:**
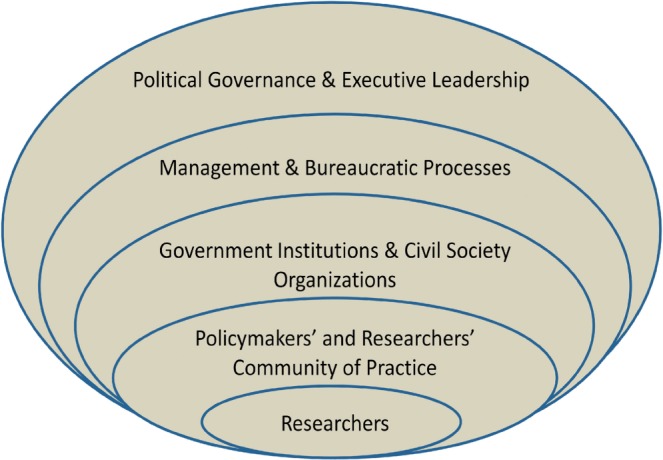
Conceptualizing health researchers’ working context: using the example of researcher push (van Kammen *et al.* 2006), and moving outwards from the ‘researchers’ oval, the researcher and/or knowledge products will have to enter into the space occupied by the community of researchers and policymakers. These communities of practice are formal or informal networks, and are found within structures of government departments and civil society organizations which are involved with management and bureaucratic processes comprising the machinery of government. These, in turn, are informed by political governance and executive leadership decisions which steer decision making.

Table 1 summarizes the facilitators and barriers to the uptake of research into policy, as noted by the Argentine researchers. They have been organized within a hierarchical structure through which evidence maneuvers to inform policy. As seen in Figure 1, the researcher, and his/her primary function of producing knowledge, is nested within an increasingly complex and broader system: communities of practice, government departments/civil society organizations, bureaucratic processes and political governance and executive leadership.

**Table 1:** Perceived facilitators and barriers to the use of research

	Facilitator	Barrier
Political level: executive leadership	Policy co-ordination between provinces	Lack of continuity between ruling governments leading to a loss of contacts/relationships between researchers and policymakers as well as a loss of experiential knowledge and a loss of institutional history
	‘Contagious effect’ where provinces look to and adopt neighbouring provinces’ policies and programmes, adapted to their own context	As a federal system there is diminished convening power and research-informed policy setting at the central level regarding provincial health systems
		Short-term thinking by policymakers
		Lack of clear policies and strategic directions
		Perception of politicians being corrupt
Management level: bureaucratic processes	Incorporating feasibility studies to enable research translation into policies and programmes	Disconnect between health research priorities and funding as a result of a lack of research accountability
		Time-bound constraints in responding to policy demands relative to research time required to answer policy question
		Loss of time in searching for research
		The inability to obtain good quality research
		The effort needed to apply the research to the particular policy issue
		The culture of the department/organization one is working within
Institutional level: Government departments and civil society	Formalization of a national research system	Diminished effectiveness of government programmes aimed to promote research use in policymaking
	At the time of this study, Argentine Forum for Health Research was identified as an institutional-level facilitator whose convening power focuses on public health research priority setting in Argentina	Few health policy analysis and research institutions outside of the government system (diminished external capacity)
Community of practice level: researchers and policymakers	Researchers should learn to generate products which are useful to policymakers (e.g. policy briefs, executive summary)	Language/vocabulary used by researchers is difficult to understand
	Personal contact/personal relationship exists between researchers and policymakers	Lack of trust between policymakers and researchers
	Inclusion of bureaucrats, programme implementers and other stakeholders in the entire research process	
	Use of the media/press to draw attention to research	
	Timing/opportunity	
	Researchers must take the initiative to share their work/input to the process and seek out ways to interact with policymakers	
Researcher level: determinants of research availability	Increased funding for research	Lack of health policy-oriented research
	Researcher belongs to an institution with longer research traditions and has a social network of colleagues/peers to draw on as resources for navigating the publishing process	Limited experience and capacity of the researchers to publish, thereby limiting available evidence; not part of the research culture
		Lack of familiarity with public health concepts due to a biomedical research focus
		Language barriers to publishing Spanish research in English mainstream science and public health journals

### Political governance and executive leadership

Informants indicated that awareness of evidence-based policymaking in Argentina is recent and that policymakers were unlikely to use evidence when developing policies. As they believed Argentine policymakers felt unaccountable to their constituents, policymakers were perceived to make public policies with ‘no rational support or with no relation to the public interest’:

There is one very important barrier (to using research results to inform policymaking), and that is that in Argentina the political system is a weak democracy. We still have a very weak democracy in Argentina. I think this is one of the strongest explanations you can find to not have evidence-based policies in Argentina.

This perception of a weak democracy may be linked to caudillismo,^1^ a cultural influence which can be understood through the immigration-induced cultural shifts in Argentina over the last century. One researcher describes migrants as having the drive to be ‘self-made men’, often choosing to come to Argentina, perceived at the time as the riskier of the migrant-receiving countries, because ‘the more there was to risk, the more successful they could become’. Two phenomena are associated with cultural influences on health policymaking in Argentina: a short-termist thinking which the informant attributed to the waves of immigrants believing they would be in Argentina only for a short time before returning to their home country, and the issue of ‘caudillismo’ and individual leadership, as opposed to collectivity:

When you begin to speak with people in public health about the big projects, they will give you names of people. Not projects. They will say: ‘Dr. …’. These are the leaders. Therefore, this caudillismo, in addition to the short-term view, and the need for individuality is very strong. Unfortunately, this individualism, in times of crisis means: ‘I save myself.’

From a national governance and public health system-building perspective, these entrepreneurial characteristics and emphasis on leadership, combined with a frequently unstable governance structure, undermine an ‘evidence-based policymaking culture’. One of the greatest barriers to research influencing policy was the lack of continuity between governments and high turnover of government staff. The policies of the previous government are overridden by the new government, new people are hired and existing contacts and relationship history are lost. It was perceived that there is rarely policy continuity when a new government takes power, that every new administration disregards historical experiences and that this is idiosyncratic of Argentine politics.

Mechanisms to ensure continuity of policies between governments, such as legislation, are perceived to not function or be as effective as they should be. Researchers suggested that ensuring continuity at the institutional level involved the provinces and the federal government working together in a co-ordinated manner. However, informants indicated that rather than following federal edicts, there is a ‘contagious effect’ where provinces often look to neighbouring provinces already having implemented a policy before deciding to implement within their own province.

A lack of clear policies and strategic directions at the executive levels was also identified as a barrier. Without clear political priorities, policymaking is hampered by a lack of clear direction and purpose, which in turn does not allow for sufficient or accurate access to research or researchers to inform the policy purpose.

### Management and bureaucratic processes

Researchers highlighted the disconnect between governmental health research priorities and institutions that fund research and that funding institutions do not hold researchers accountable to adhering to research priorities:

The truth is that CONICET [el Consejo Nacional de Investigaciones Científicas y Técnicas] asks me, as a researcher [funded by CONICET] that I every year, and then every two years, give them an account of that which I am doing … The truth is I could do research on a very specific topic that is not a health priority. And nobody would tell me I couldn’t do it.

Incorporating feasibility studies into the research was one option that was proposed for diminishing barriers in research translation into policy and programmes, as was mandating that research proposals include systematic protocols for research transfer*.* Other process barriers can be found in Table 1.

### Government institutions and civil society organizations

At the time of this research, the federal government had taken steps to formalizing a national health research system through the creation of an extra-departmental programme to facilitate the uptake of research into policy. Although some researchers perceived it to be where research priorities are decided, the effectiveness of ‘Salud Investiga’ (Researching Health) was questioned:

A structure such as Salud Investiga which is just making its way into a Ministry that has other departments that have been around and working for years, well, it means that it is undergoing a major power struggle.

Researchers indicated that there were few institutions doing public health research. There were three institutions identified by informants that were perceived to ‘do serious research’, are relatively independent of government and have the opportunities to translate research into policy. These were the Centro de Implementación de Políticas Públicas para la Equidad y el Crecimiento, the Instituto de Efectividad Clínica y Sanitaria and the Centro de Estudios de Estado y Sociedad.

Researchers at NGOs believed their role is to produce high-quality research, train individuals to ensure a supply of human resources for health research and create a community of researchers. It was believed by informants that research NGOs met the researchers’ responsibilities to society and collective action in Argentina through advocacy efforts and using research to support policy decisions.

The scarcity of NGOs/health policy research and analysis institutions working on public health and health systems advocacy issues in Argentina was identified as a barrier to using research to inform policies. Historical reasons, government edicts and dictatorships and a lack of funding and other support have limited NGO development and opportunities to engage intellectually with policymakers, leading to a lack of ‘spaces for dialogue’.

### Community of practice: relationships between health researchers and policymakers

The extent of researchers’ experience with policymakers varied from having little experience to others being professionally trained in State institutions and being advised by politicians and government workers. Others had been policymakers themselves, with several informants having been provincial Ministers of Health at one time during their careers.

The challenges faced between researchers and policymakers’ communities of practice included policymakers perceiving researchers as proud, while some researchers had a deeply negative view of politicians and budget administrators, linked to corruption. Argentine politicians are perceived as being improvisers rather than planners, the reason most frequently attributed by informants for the failure in using research in policymaking. Researchers also indicated that it was mainly by luck and coincidence if research was taken into account in policymaking. Moreover, the political context was inescapable:

… the moment I started working in rural areas, [I began] to experience the lack of response by policy decision-makers, or their lack of vision. I wanted to be with the people, not politicians, and that is when one of my colleagues said “You are in public health and public health is political.” So you cannot avoid policymakers because you’ll have to be permanently in contact with them because they are actors.

Researchers believed that policymakers and their advisors lacked technical capacity and knowledge about health issues affecting the Argentine people. Informants recognized that it was difficult to complement technical and political expertise within a single decision maker and that his/her team was meant to have technical expertise:

Behind a bureaucrat, there is always a technical expert who justifies that which the bureaucrat is doing … The problem is the [level and quality] of training behind this bureaucrat.

Research uptake into policymaking was facilitated when the decision maker solicited and was involved in the research. Including ‘efectores’^2^ in the research project was believed to catalyze research translation into policies. This was the strategic and deliberate approach taken by one informant who reported the inclusion of programme managers and stakeholders from the beginning of the study:

We designed [a clinical trial], together thought about the research aspects, and executed it. At the moment of deciding if this strategy will be adopted, you also have to decide who will be financing it — the political decision-maker — a sub-Secretary [like a vice-minister or deputy minister], one who is not a technical expert. At this level it is much easier to accept that the strategy is valid because it is being presented by a group of researchers, as well as his/her own employees, the one who will be implementing it. With both sides presenting, the research results and recommendations will be implemented.

Policymakers were perceived to trust research results more when a member of their department was linked to the research project. A more trusting relationship helps facilitate the uptake of research into policymaking, with relationship- and trust-building identified as key strategies in working with policymakers:

… you have to develop a relationship with [policymakers] and make the person feel that they can trust you. So probably the politician feels very exposed to be working in a public scenario, they need people they can trust, so it’s a long-term investment … and perhaps you have success, but your contact may end up leaving the public service, so you win a friend but have lost an opportunity to work on public policy.

The dynamic of differing professional mandates poses challenges for researcher–policymaker working relationships. The role of the researcher is to question and consider numerous options and varying factors, which relays uncertainty to policymakers. This approach is opposite of the certainty and decisiveness required of the politician:

The politician also requires certainty … Often I have seen the desperation on the faces of a politician, when you are asked something and respond ‘It depends’ and start to give all the variables … And what he wants is a single answer … And you are speaking to him in terms of probability and the other person hears it in terms of certainty, in terms of truth.

Researchers noted that they were ‘imprisoned’ by critical and analytical language, which is difficult to understand by the policymaker. Language constraints and vocabulary use seem to be professionally linked: one informant commented that when they became a Minister, they found that they had adopted the speech and behaviour of a decision maker, even when speaking with researchers.

It was believed to be important for researchers to take initiative, involve themselves in policy councils and seek out ways to interact with policymakers. Researchers indicated that the researchers themselves should learn to generate products, such as policy briefs or executive summaries accompanying reports, seen to enable policymakers’ use of research.

Researchers also suggested enhancing decision makers’ capacity through short training sessions to help policymakers learn how to use research to make decisions, or developing higher schools of government, or public health training institutes for government officials to train individuals in both points of view.

### Determinants of research availability

Researchers identified that their own publishing capacity was limited. Relative to the academically rigorous level required to publish in international journals, the majority of research projects in Argentina was perceived to be small and not rigorous or in-depth enough to warrant international journal standards. Challenges to publishing included translating from Spanish to English, as well as lack of a ‘research publishing culture’:

You have seen that Argentina publishes very little research in international journals. There are very few researchers who publish in international journals. Because the people are not used to writing in Argentina. They write very little.

One informant discussed his work on the Argentine research landscape which showed that the majority of publications belong to institutions with longer publishing traditions. In speaking to researchers in smaller institutions, those researchers felt more isolated and alone, without experience or support, leading to a lack of motivation for publishing.

Researchers noted that when there is a mass of researchers together in one place, it stimulates the development of research and promotes interaction and contacts between researchers (Table 1). This may also be what helps drive the differences in the proportion of ‘successful’ researchers working in the provinces compared with the city of Buenos Aires:

The reality is that Buenos Aires had practically 70% of the research capacity of the country. The critical mass of research and resources is here in Buenos Aires.

Researchers reported that there are disproportionate amounts of research funds going to the federal capital of Buenos Aires. This is perceived to be mainly due to a ‘leadership bias’ in scholarship allocation. Because so many research leaders are based out of Buenos Aires, the federal capital receives a higher proportion of scholarships and research funding compared with the provinces.

## Discussion

Figure 1 demonstrates the schematic representation of the context within which Argentine researchers work. It delineates the organizational and procedural levels that evidence must negotiate when informing policy. The use of the researcher’s research and knowledge production occurs via consumption by individuals such as other researchers and policymakers (in the oval signifying a community of practice), who behave according to institutional norms of their culture and professional mandate, which are housed within different organizations at the societal level (the oval representing government institutions and civil society organizations). Consequently, bureaucratic processes must be managed before research and its use in policymaking can be conciliated with executive decision-making and political processes. In addition to researcher push, Figure 1 is also useful for representing ‘user-pull’ (van Kammen *et al.* 2006), if beginning from the outermost layer and moving inwards.

This organizing structure was developed based on themes emerging from the interviews. These themes were organizationally similar to those that Trostle *et al.* (1999) discovered in Mexico 14 years earlier, with emphases on content, actors, process and context (Trostle *et al.* 1999). Our conceptualization is different in that it places research producers, and not policies, at the center of the model. This is appropriate given our goal of understanding the context in which the researcher operates, and the levels of societal organization that must be manoeuvred for evidence to influence policies. Our organizational framework may be conceptually useful at the level of government, policy departments, civil society organizations, programme implementers and also researchers, in identifying actors involved in the research-to-policy process, and when determining which barriers and facilitators exist and to troubleshoot any potential hindrances that may occur.

Support for this conceptualization can be seen in Koon *et al.*’s recent publication around embedding health policy and systems research in decision making (Koon *et al.* 2013). The work undertaken in Argentina helps to provide a specific model for the country context in which to test the macro-level theories laid out in Koon *et al.*’s work as they relate to national health research systems. In addition, the work in Argentina helps to provide support to Koon *et al.*’s findings that the extent that an organization was connected to others, as well as to decision makers matters more than the quantity and quality of research generated. At a country level, in the absence of formal health policy and research institutions, and as noted in the results, the importance of research leaders (caudillos) and their connections to decision makers were also perceived as important factors in research uptake by decision-makers.

### Facilitators and barriers to evidence-informing policymaking

Studies looking at evidence-based policymaking in LMICs cover a wide spectrum of health issues and policies and a large number of countries and geographic regions, such as Burkina-Faso (Gerhardus *et al.* 2000), Mexico (Trostle *et al.* 1999), Pakistan (Hilderbrand *et al.* 2000) and South Africa (Moodley and Jacobs 2000). Overlapping facilitators found in those studies and in this one (Table 1) included the importance of timing, the use of research summaries, the use of the media to raise awareness about research results, informal relationships and personal contacts with policymakers and the importance of advocacy efforts on the part of the researcher. Unique to Argentina at the time of data collection was the institutional-level attempt to redress the disconnect between research funding and health priorities by creating programmes and institutions to help align research priorities with health priorities, such as the Argentine Forum for Health Research or Salud Investiga.

Although the legislative structure of Argentina invites provinces to adhere to national laws, the provinces hold most of the power in public health policymaking and decide which law they will promote and which national programme will be allowed to be implemented in their provinces. This behaviour of policy adoption based on neighboring province’s precedent implies that a formal or informal policy diffusion network exists.

Barriers that were identified by Argentine researchers (Table 1) and also appeared in the aforementioned studies include the extent of support for biomedical vs social research as well as a lack of interaction between researchers and decision makers. Other barriers identified included a lack of continuity between ruling governments, leading to a loss of contacts, disruption of relationships, as well as a potential lack of familiarity with the state of knowledge related to the health policy issues, accompanied by a loss of institutional memory.

Researchers’ publication potential was limited by few resources being available to hire translators or correct the English versions of Spanish research. However, the lack of a social network was also a limiting factor. Young Argentine researchers and students are rarely exposed or trained to publish in international journals. These limitations may contribute to the identified lack of a publication culture.

Although ‘trust’ was not hypothesized to be an important theme prior to the interviews, it unexpectedly emerged strongly and consistently when discussing researchers’ perceptions of policymakers and the use of evidence, and linked to a lack of confidence in policymakers. Gilson (2003) argues that ‘trust underpins the co-operation within health systems that is necessary to health production, and that a trust-based health system can make an important contribution to building value in society’. Gilson (2003) also argues that health systems are inherently relational and so many of the most critical challenges for health systems are relationship and behaviour problems; the role of trust is in facilitating collective action and co-operation among people to achieve common goals. Furthermore, Thiede (2005) argues that health policy’s obligations in securing access go beyond the supply of health care, including information, and that trust assumes a key position within this transactional process of information exchange or communicative interaction. The recurrent theme around trust and ‘lack of trust’ in the interviews and its emergence in the analysis and role within a research system lead us to postulate that Gilson’s and Thiede’s argument is applicable to health research systems and research production. In the Argentine context, political barriers are historically linked to the sociopolitical impact of military dictatorships and cycles of economic recessions, and trust in the context of Argentine history merited further investigation into the sociopolitical context.

### Trust and Argentine history

Trust as a facilitator of research uptake is a challenging characteristic to address within the Argentine context of political volatility, changing governments and high staff turnover. Argentina’s tumultuous political history directly affected the academic and research communities, with effects being felt to this day. The military coup of 24 March 1976 installed a repressive dictatorship that ruled during a period in Argentine history known as ‘The Dirty War’. A programme of state terrorism ran from 1976 to 1983 aimed to eliminate political dissidents, where the military utilized kidnappings, torture, killings, targeted assassinations and disappearances to impose a reign of terror and a culture of fear; it is estimated that up to 30 000 persons from that era remain ‘disappeared’ (Kaiser 2002). The disappeared included students, researchers and professors as well as journalists, trade unionists and Peronist and Marxist guerrillas (Banks *et al.* 2010). Mass resignations of researchers and professors at the University of Buenos Aires in protest of the dictatorship are believed to have catalyzed the deterioration of the public university system and fracture of the research community.

Researchers’ distrust towards policymakers and even other researchers are linked inextricably to the sociopolitical history of Argentina, which contributed to shaping researchers’ identities in opposition to policymakers. The researchers interviewed lived their formative years through this period, and had been graduate students or just beginning their careers. Despite expectations that justice would be served upon the restoration of democracy, a combination of factors, ranging from political opportunism to military pressure, resulted in politicians’ promises to re-establish justice remaining unfulfilled (Kaiser 2002).

The people’s trust in politicians and policymakers continued to be eroded, with people’s scepticism in politicians peaking with the economic crisis in 2001 (Cleary and Stokes 2006, p. 91). The Argentine government froze citizens’ bank accounts, and hyperinflation and currency devaluation severely affected the standard of living and quality of life of many Argentines. This was followed in early 2002 by an economic collapse.

Although some researchers theorize that scepticism of politicians, and not trust, is the better attribute of a healthy democracy (Cleary and Stokes 2006), in this study, we found that researchers’ lack of trust was considered to be a barrier to the use of research in policymaking, as it undermined relationships/personal contact between researchers and policymakers, a factor which has been found to be a facilitator to evidence-based policymaking in other studies (Innvaer *et al.* 2002).

### Interpretations of research evidence

The researchers we interviewed highlighted the importance of ‘context’—that evidence cannot, in isolation, inform policies, but must be interpreted and used within a certain context. This has been echoed in the work done by Lewin *et al.* (2009) on ‘evidence about local conditions’, which is evidence that is available from the specific setting(s) in which a decision or action on a policy or programme option will be taken. These authors argue that such evidence is always needed, together with other forms of evidence, in order to inform decisions about options (Lewin *et al.* 2009).

### Opportunities for researchers, policymakers and donors

For Argentine health researchers, and researchers studying evidence-informed policymaking and the role of researchers within the wider health system, this work is a foundational piece for future research and to develop opportunities for local health researchers to inform policy in Argentina. It also presents an opportunity to follow-up on this ‘baseline’ work and determine the extent by which institutionalization of research uptake has succeeded and altered the Argentine health policy landscape. Recent work on measuring trust in health systems (Ozawa and Sripad 2013) may also offer opportunities for researchers to further investigate and measure trust within the context of research uptake in policymaking.

For policymakers and in-country research councils and funders, this article provides a deeper understanding of researcher perceptions. This understanding can inform and improve programme design when developing interventions to enhance research utilization in order to develop fair and rational health policies. For example, accountability processes could be put in place to ensure that research funding is addressing national health priorities. The misalignment between health priorities and research undertaken in Argentina further contributes to research inefficiencies as limited resources are not being optimally used to address health priorities in Argentina. Research-funding bodies should mandate that researchers include a realistic and applicable research implementation plan when submitting research proposals, and when policy applicable. The policy implications of the research should be clearly outlined by the researcher so as to facilitate policymakers’ understanding of the implications of the research results. Additionally, the value added of research activities can be improved by linking funding to health research priorities and/or research being undertaken to answer specific policy questions. It may also help facilitate ‘deliberative processes and meaningful partnerships between researchers and users that help them to jointly ask and answer relevant questions’ (Moat and Lavis 2013).

For donors and development agencies interested in health research capacity building, this research demonstrates a need for investment in building capacity and training health researchers to interact with the public policy world and enhancing research communications and transferability to decision makers. It also highlights an opportunity to invest in implementation research platforms, such as health policy research and analysis institutions, which could serve as knowledge brokers between researchers and policymakers (Bennett *et al.* 2012). This in turn would help strengthen national health research systems, and their contribution to strengthening health systems.

### Limitations

This study was based on a sample of individuals who were interested in participating and agreed to speak with us. Therefore, this snowball sample is limited by non-random selection procedures, correlations between network size and selection probabilities and reliance on the subjective judgements of informants (Johnson 2005). Furthermore, it is difficult to draw definitive conclusions from the findings or at least generalize them to larger groups.

As Spanish is a high-context language, translating themes and quotes into English required altering the coding methodology from the originally planned line-by-line coding to thematic identification. The context of the phrasing was especially carefully regarded. For example, ‘los politicos’ referred to elected politicians, but the term was used interchangeably with decisores (decision makers), given that they work in government. In short, the more appropriate definition and translation of the word politiques (French) or ‘políticas’ (Spanish) was taken to mean policy or politics given the context and conversation in which the word is used, taking into account that the people using it may also not distinguish the term for one or the other in their use.

From a sociological and anthropological standpoint, language and culture tend to be intertwined. In addition to demands for government accountability and transparency, and an empowered population (Davies *et al.* 2000), one of the reasons why evidence-based decision making has gained importance in the UK, Canada and Australia (and now increasingly in the USA) may be that the English language, being relatively low-context, allows for an easier transfer and use of research into policies.

Although the time lapse between data collection and publication may be perceived as a limitation, the organized framework contextualizing the Argentine researchers’ work environment is based on data collected from health researchers from a wide variety of biological and social science academic backgrounds, working in universities, in a combined research and decision-making capacity for provincial Ministries of Health, or within NGOs and think tanks. The researchers’ experiences with informing health policies or programmes with their research, and in working with policymakers in the Argentine public health sector, irrespective of content area or local, provincial or national scale, based on their lifetime of research, provides a strong foundation for the analysis and interpretation of results that remains theoretically current.

## Conclusion

The uniqueness of this research is its focus on the supply side of the research-to-policy spectrum in a middle-income country, that it places researchers at the centre of the study, it explores their role in the evidence-informed decision making and proposes a new organizing schematic for thinking about how researchers interact with (and can influence) their working environment. Past LMIC-focused research has centred on the decision makers, whereas less is known about the national health research systems LMIC context in which the researchers navigate, nor how researchers perceive their environment and work as related to policymaking. The proposed framework for conceptualizing the health researchers’ working context provides insight into the organization of national health research systems’ for those outside of Argentina, and may be relevant to their countries’ research systems. In addition, this work sheds light on the importance of developing a ‘research publishing culture’ within a research system as a determinant of access to research availability, highlights the importance of trust in health research systems and identifies the caudillo effect and its relationship to evidence-informing policymaking. Further research is needed to provide greater insight on the supply side of the research availability and the role of the national health research system to inform policymaking in other LMICs.

## Supplementary Material

Supplementary Data

## Endnotes

^1^ In Latin America, caudillismo is the practice of glorifying a single leader and caudillo is a term used to refer to charismatic populist leaders.

^2^ Efectores were defined by the researchers as individuals responsible for public health implementation, and included Ministries of Health at the national and provincial levels; consumers of the research results and implementers of the research findings (including programme managers and policymakers).

## Appendix 1 Interview guide—English

Exploring perceptions of the research-to-policy process and evidence-based policymaking through interviews with Argentine health researchers

**Table:**

Theme	Personal motivations for conducting research
Potential Questions	How did you become interested in doing research?
	What sustains your interest in doing research?
	What aspects of research interest you?
	(e.g. Interactions with researchers doing the research; Reading and discussing research reports or articles; Ensuring access to essential health interventions for the poor and for ethnic and religious minorities; Benefits of the research to research participants)
	What do you see as incentives for doing research? What motivates you?
	What are some disincentives in doing research?

Theme	Overall perception of policy and policymakers
Potential Questions	How do you think about policy? How would you define it?
	Can you tell me a little bit about the role policy plays in your life, as it pertains to your research?
	How would you define a policymaker?
	Have you had any direct or indirect experiences with policymakers?
	Have you collaborated with policy-makers?
	What do you see as the role of policy-makers in the field of international health?

Theme	Barriers to use of research
	What do you see as the barriers to using research results to inform policy-making?

Theme	Facilitators to use of research
	What do you think would facilitate using research results to inform policy-making?

Theme	Promotion of evidence: researcher’s responsibilities
Potential Questions	What do you see as the purpose of your research?
	What do you see as your role in achieving that purpose?
	What is research to you? Do you distinguish between different kinds of research? Do different types of research (e.g. lab work, qualitative research, etc.) have different values to you?
	Have you ever been invited to be a member of a committee or panel in your subject area?
	Have you been in a decision-making position? Have you had to formulate policies?

Theme	Knowledge translation
Potential Questions	What do you think is the most effective way of influencing policy?
	Is there a particular mechanism you see as critical for your research informing policy?
	What do you see the relationship being between researchers and policymakers?
	Do you want to see your research implemented in practice? Through what process do you see it being implemented?
	What are the principle means of supporting your research? Have there been any plans for implementation with respect to any of these grants/awards?
