# Supplementary material for: Exploring health researchers’ perceptions of policymaking in Argentina: a qualitative study
Source: Health Policy Plan. 2014 Sep 11;29(Suppl 2):ii40–9. doi: 10.1093/heapol/czu071 (PMC4202917; doi:10.1093/heapol/czu071)
Supplement: Supplementary Data [file supp_29_suppl-2_ii40__index.html]

Exploring health researchers’ perceptions of policymaking in Argentina: a qualitative study — Supplementary Data 

# Exploring health researchers’ perceptions of policymaking in Argentina: a qualitative study

## Supplementary Data

files

**Files in this Data Supplement:**

- Supplementary Data - rtf file
- Supplementary Data - rtf file
- Supplementary Data - rtf file
