# Supplementary material for: Exploring health researchers’ perceptions of policymaking in Argentina: a qualitative study
Source: Health Policy Plan. 2014 Sep 11;29(Suppl 2):ii40–9. doi: 10.1093/heapol/czu071 (PMC4202917; doi:10.1093/heapol/czu071)
Supplement: Supplementary Data [file supp_czu071_Appendix_1_Interview_Guide_English.rtf]

Appendix 1: Interview Guide – English

Exploring perceptions of the research-to-policy process and evidence-based policy-making through interviews with Argentine health researchers 


Theme	Personal motivations for conducting research
	
Potential Questions	How did you become interested in doing research?	
	What sustains your interest in interest?
What aspects of research interest you? 
(e.g. Interactions with researchers doing the research; Reading and discussing research reports or articles; Ensuring access to essential health interventions for the poor and for ethnic and religious minorities; Benefits of the research to research participants)	
	What do you see as incentives for doing research?  What motivates you?  	
	What are some disincentives in doing research?	


Theme	Overall perception of policy and policymakers
	
Potential Questions	How do you think about policy?  How would you define it?	
	Can you tell me a little bit about the role policy plays in your life, as it pertains to your research?	
	How would you define a policymaker?	
	Have you had any direct or indirect experiences with policymakers?
Have you collaborated with policy-makers?  	
	What do you see as the role of policy-makers in the field of international health?	


Theme	Barriers to use of research	
	What do you see as the barriers to using research results to inform policy-making?  	


Theme	Facilitators to use of research	
	What do you think would facilitate using research results to inform policy-making?	


Theme	Promotion of evidence: researcher's responsibilities
	
Potential Questions	What do you see as the purpose of your research?	
	What do you see as your role in achieving that purpose?	
	What is research to you?  Do you distinguish between different kinds of research?  Do different types of research (e.g. lab work, qualitative research, etc.) have different values to you?	
	Have you ever been invited to be a member of a committee or panel in your subject area?	
	Have you been in a decision-making position?  Have you had to formulate policies?	


Theme	Knowledge translation
	
Potential Questions	What do you think is the most effective way of influencing policy?	
	Is there a particular mechanism you see as critical for your research informing policy?	
	What do you see the relationship being between researchers and policymakers?	
	Do you want to see your research implemented in practice?  Through what process do you see it being implemented?	
	What are the principle means of supporting your research?  Have there been any plans for implementation with respect to any of these grants/awards?	
