# Supplementary material for: Exploring health researchers’ perceptions of policymaking in Argentina: a qualitative study
Source: Health Policy Plan. 2014 Sep 11;29(Suppl 2):ii40–9. doi: 10.1093/heapol/czu071 (PMC4202917; doi:10.1093/heapol/czu071)
Supplement: Supplementary Data [file supp_czu071_Figure_1_Health_Researchers_Working_Context.rtf]

Figure 1: Conceptualizing Health Researchers' Working Context


Using the example of 'researcher-push' (van Kammen et al., 2006), and moving outwards from the 'Researchers' oval, the researcher and/or knowledge products will have to enter into the space occupied by the community of researchers and policymakers.  These communities of practice are formal or informal networks, and are found within structures of government departments and civil society organizations which are involved with management and bureaucratic processes comprising the machinery of government.  These, in turn, are informed by political governance and executive leadership decisions which steer decision-making.
  
