# Supplementary material for: Exploring health researchers’ perceptions of policymaking in Argentina: a qualitative study
Source: Health Policy Plan. 2014 Sep 11;29(Suppl 2):ii40–9. doi: 10.1093/heapol/czu071 (PMC4202917; doi:10.1093/heapol/czu071)
Supplement: Supplementary Data [file supp_czu071_Table_1_Facilitators_and_Barriers_to_the_Use_of_Research.rtf]

Table 1: Perceived Facilitators and Barriers to the Use of Research
	Facilitator	Barrier	
Political Level: Executive Leadership	·	Policy coordination between provinces	·	Lack of continuity between ruling governments leading to a loss of contacts/relationships between researchers and policymakers as well as a loss of experiential knowledge and a loss of institutional history	
	·	“Contagious effect” where provinces look to and adopt neighbouring provinces' policies and programs, adapted to their own context 	·	As a federal system there is diminished convening power and research-informed policy-setting at the central level regarding provincial health systems	
		·	Short-term thinking by policymakers	
		·	Lack of clear policies and strategic directions	
		·	Perception of politicians being corrupt	
			
Management Level: Bureaucratic Processes	·	Incorporating feasibility studies to enable research translation into policies and programs	·	Disconnect between health research priorities and funding as a result of a lack of research accountability	
		·	Time-bound constraints in responding to policy demands relative to research time required to answer policy question	
		·	Loss of time in searching for research	
		·	The inability to obtain good quality research	
		·	The effort needed to apply the research to the particular policy issue	
		·	The culture of the department/organization one is working within	


			
Institutional Level: Government Departments and Civil Society	·	Formalization of a national research system	·	Diminished effectiveness of government programs aimed to promote research use in policymaking	
	·	At the time of this study, FISA (Argentine Forum for Health Research) was identified as an institutional-level facilitator whose convening power focuses on public health research priority-setting in Argentina	·	Few health policy analysis and research institutions outside of the government system (diminished external capacity)	
			
Community of Practice Level: Researchers and Policymakers	·	Researchers should learn to generate products which are useful to policymakers (e.g. policy briefs, executive summary)	·	Language/vocabulary used by researchers is difficult to understand	
	·	Personal contact/personal relationship exists between researchers and policymakers	·	Lack of trust between policymakers and researchers	
	·	Inclusion of bureaucrats, program implementers and other stakeholders in the entire research process		
	·	Use of the media/press to draw attention to research		
	·	Timing/opportunity		
	·	Researchers must take the initiative to share their work/input to the process and seek out ways to interact with policymakers		
			
Researcher Level: Determinants of Research Availability	·	Increased funding for research	·	Lack of health policy-oriented research	
	·	Researcher belongs to an institution with longer research traditions and has a social network of colleagues/peers to draw on as resources for navigating the publishing process	·	Limited experience and capacity of the researchers to publish, thereby limiting available evidence; not part of the research culture	
		·	Lack of familiarity with public health concepts due to a biomedical research focus	
		·	Language barriers to publishing Spanish research in English mainstream science and public health journals	
